# Proteomic analysis of neurons microdissected from formalin-fixed, paraffin-embedded Alzheimer’s disease brain tissue

**DOI:** 10.1038/srep15456

**Published:** 2015-10-21

**Authors:** Eleanor S Drummond, Shruti Nayak, Beatrix Ueberheide, Thomas Wisniewski

**Affiliations:** 1 Department of Neurology, NYU Langone Medical Center, New York, NY, USA; 2 Proteomics Resource Center, Office of Collaborative Science, New York University School of Medicine, New York, NY, USA; 3 Department of Biochemistry and Molecular Pharmacology, NYU Langone Medical Center, New York, NY, USA; 4 Departments of Neurology, Pathology and Psychiatry, NYU Langone Medical Center, New York, NY, USA

## Abstract

The vast majority of human tissue specimens are formalin-fixed, paraffin embedded (FFPE) archival samples, making this type of tissue a potential gold mine for medical research. It is now accepted that proteomics can be done using FFPE tissue and can generate similar results as snap-frozen tissue. However, the current methodology requires a large amount of starting protein, limiting the questions that can be answered in these types of proteomics studies and making cell-type specific proteomics studies difficult. Cell-type specific proteomics has the potential to greatly enhance understanding of cell functioning in both normal and disease states. Therefore, here we describe a new method that allows localized proteomics on individual cell populations isolated from FFPE tissue sections using laser capture microdissection. To demonstrate this technique we microdissected neurons from archived tissue blocks of the temporal cortex from patients with Alzheimer’s disease. Using this method we identified over 400 proteins in microdissected neurons; on average 78% that were neuronal and 50% that were associated with Alzheimer’s disease. Therefore, this technique is able to provide accurate and meaningful data and has great potential for any future study that wishes to perform localized proteomics using very small amounts of archived FFPE tissue.

Traditionally it has been difficult to perform cell-type specific proteomics using formalin-fixed paraffin embedded (FFPE) tissue sections due to the technical difficulty associated with isolating specific cell populations from tissue sections and obtaining sufficient amounts of total protein. Recent advances in proteomics methods have shown that mass spectrometry can be successfully performed using FFPE tissue^1^,^2^,^3^; however, the majority of these studies have analyzed large regions of tissue rather than individual cell populations. The ability to perform cell-type specific proteomics would help answer more targeted questions about disease, particularly neurodegenerative diseases which are known to affect very specific types of cells in discrete brain regions. Therefore, the aim of this study was to develop a method to perform quantitative LC-MS on a specific population of neurons isolated from archived FFPE brain sections using laser capture microdissection (LCM). A limited number of studies have performed LC-MS on specific cell populations isolated using LCM; however, these suggested use of fresh tissue is required and FFPE sections are not compatible for this approach^4^,^5^. For this study the examined cell population was neurons isolated from the temporal cortex from patients who had severe Alzheimer’s disease. While this technique is of great interest for any study that uses FFPE tissue, we chose to examine neurons because of the potential use of this technique to examine the basis of selective vulnerability in neurodegenerative diseases. As this was the first time LC-MS had been performed on individual cells isolated from FFPE brain tissue, initial experiments were performed to determine the optimal lysis and staining method to detect the maximum number of proteins by LC-MS. We then determined the minimal number of microdissected neurons necessary to perform quantitative LC-MS. Here, we present a strategy that allows unbiased quantification of hundreds of proteins in a specific population of neurons isolated from archived FFPE tissue.

## Results

**Figure 1:**
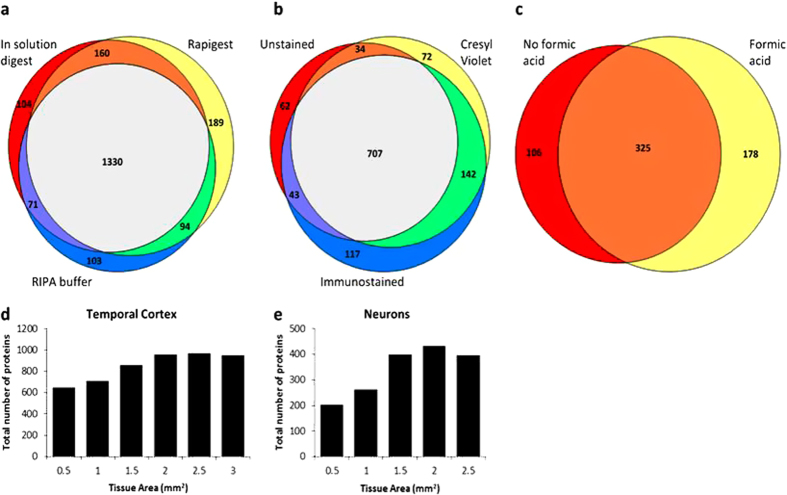
Total number of proteins detected using LC-MS comparing (a) three different lysis methods (b) three different staining methods keeping the lysis method constant (in solution digestion) (c) formic acid digestion on isolated neurons. Bar graph of total number of proteins detected by tissue area in (**d**) whole regions of temporal cortex and (**e**) neurons microdissected from the temporal cortex.

First, specific regions or cell types were isolated using LCM. This technique allows the dissection of very small areas of tissue (such as single cells) from tissue sections using a precise laser, while leaving the protein content inside cells unaffected. 8 μm sections were used throughout this study to reduce the likelihood of collection of surrounding non-neuronal tissue. Isolated tissue was then processed for LC-MS using a method modified from Alkhas *et al*.^6^. In-solution digestion without detergent, in-solution digestion with Rapigest (mass spectrometry compatible detergent) and solubilization in RIPA buffer followed by in-gel digestion were initially compared to determine the optimal sample preparation method. For these optimization experiments we used a total area of 10 mm^2^ of temporal cortex, equivalent to approximately 80,000 neurons based on our experience with this type of tissue. It was found that all three sample preparation methods detected a similar number and population of proteins, therefore showing that detergent did not improve protein detection and can be omitted in the interest of limiting sample loss due to detergent clean up (Fig. 1a; supplementary Figure 1). Therefore, all future studies were done using direct digestion without detergent to keep sample handling at a minimum. Next, as cell populations of interest can only be identified using either histological staining or immunohistochemistry, it was necessary to determine if tissue staining prior to LCM affected the total number of proteins detected. Neither cresyl violet staining nor immunohistochemistry reduced the total number of proteins detected using LC-MS (Fig. 1b; supplementary Figure 2). Furthermore, it is noteworthy that we used a typical immunohistochemistry protocol that included an overnight incubation with primary antibody, showing that a rapid immunostaining method was not necessary to maintain protein integrity.

The next aim of this study was to determine the minimal area of microdissected tissue necessary to perform quantitative LC-MS. LC-MS was performed on regions of temporal cortex ranging in size from 0.5 mm^2^ to 3 mm^2^. The total number of proteins identified reached a plateau at approximately 2 mm^2^, detecting 956 proteins (Fig. 1d; supplementary Figure 3). We then performed a similar experiment to determine the optimal amount of LCM-isolated neurons that were necessary to perform LC-MS. Neurons were collected ranging in total area from 0.5 mm^2^ (approximately 4,000 neurons) to 2.5 mm^2^ (approximately 20,000 neurons). It was found that a combined tissue area of 1.5 mm^2^ provided an adequate amount of starting protein to do LC-MS, detecting 399 proteins (Fig. 1e, supplementary Figure 4). To determine if proteins identified in microdissected neurons were typical of a neuronal population, a compilation of published proteins known to be located within human neurons was developed combining results from all previous proteomic studies using a pure human neuron population^7^,^8^,^9^,^10^,^11^,^12^,^13^. In addition, we made a separate less stringent database that also included all proteins identified using the keywords “neuron” and “neuronal” in the UniProt human database (accessed 9/4/14). 306 of 399 proteins (77%) detected in our neuronal sample were confirmed neuronal proteins using the more stringent database, suggesting that the array of proteins detected in microdissected neurons was reliable (Supplementary Figure 4). A similar method was also used to determine if the proteins that were detected in our neuronal sample had been previously associated with AD. A second compilation of AD-associated proteins was established that combined the results from studies that identified proteins in neurofibrillary tangles^7^,^8^,^9^, proteins up-regulated in plaques^14^, proteins that had significantly altered expression in various regions and/or fractions of the AD brain in comparison to control brains^10^,^15^,^16^,^17^,^18^,^19^,^20^,^21^,^22^,^23^,^24^,^25^ and all proteins that contained the keyword “Alzheimer” in the UniProt human database (accessed 9/4/14). It was found that 209 of our total identified 399 proteins (52%) had previously been associated with AD (supplementary Figure 4). This shows that our results are consistent with previously published studies; however it is important to note that the generated databases are not an exhaustive list and are only a starting point that will continue to expand once further studies, such as ours, are done.

A characteristic feature of AD, like many other neurodegenerative diseases, is the accumulation of insoluble aggregates of proteins. Therefore, we collected an additional neuronal sample (2 mm^2^ area, approximately 16,000 neurons) and solubilized the tissue with 70% formic acid to determine if this permitted the detection of additional insoluble proteins. Formic acid treatment increased the total number of proteins detected compared to the 2 mm^2^ area with in-solution digestion alone (Table 1; 503 vs. 431) and 325 proteins were common in both samples (fig. 1c). The majority of unique proteins detected in each sample were of low abundance (Peptide spectral matches (PSM) <5; supplementary Figure 5) indicating that formic acid treatment did not prevent the detection of abundant soluble proteins. However, formic acid treatment generally decreased the number of peptides per protein detected. The exception was 37 proteins that showed greater sequence coverage after formic acid treatment (as determined by a PSM increase >5 compared to in-solution digestion; supplementary Figure 5), 13 of which were uniquely detected. Importantly, one protein that was only detected after formic acid treatment was isoform-D of tau, indicating that it is only present in the neuronal soma in an insoluble state. Interestingly, no isoforms of tau were detected after direct digestion in neurons, but multiple isoforms were detected in whole regions of the temporal cortex (supplementary Figures 1,2 and 3). Thus, suggesting that soluble tau was only present in the cortex outside the cell soma.

## Discussion

We have described a new method that allows the identification of hundreds of proteins from localized cell populations isolated from FFPE tissue. To date, there have been four previous studies that have performed proteomics on neurons isolated from human brain sections using LCM^5^,^8^,^9^,^10^. Importantly, these previous studies have only been successful using frozen tissue, not FFPE tissue. The method we have described is a significant improvement from these previous studies because we have not only detected more proteins, but have done so using FFPE tissue rather than frozen tissue.

Previous studies have been hesitant to use FFPE tissue for proteomics because of concerns about formaldehyde-induced modifications of proteins and degradation of proteins during tissue processing^4^,^5^. As a result, snap-frozen tissue is still considered the gold standard for proteomics studies, however there is only very limited amounts of human snap-frozen tissue available for research. Therefore increasing numbers of studies have examined the potential of using more widely available FFPE tissue for proteomics studies. Encouragingly, it has been shown in multiple studies that similar proteomics data can be generated from FFPE and snap-frozen tissue^26^,^27^,^28^,^29^,^30^,^31^,^32^,^33^,^34^,^35^,^36^,^37^,^38^,^39^,^40^,^41^,^42^. Overall these studies found that the use of snap-frozen tissue resulted in slightly greater protein detection than FFPE, but that the majority of proteins detected in FFPE tissue were similarly detected in frozen tissue. Importantly, it was also noted that FFPE processing did not significantly alter the subcellular or molecular function gene ontology distribution for detected proteins, showing that FFPE processing did not prevent detection of specific populations of proteins. The major determinant of whether successful proteomics data can be generated from FFPE tissue appears to be the type of sample preparation done prior to LC-MS. Indeed, we found that tissue heating prior to trypsin digestion was essential to allow the extraction of proteins from our very small samples and the consequent identification using LC-MS. In fact, we found heat treatment prior to RIPA buffer lysis and in-gel digestion resulted in 8 times more proteins being identified in comparison to identical samples that did not receive heat treatment (data not shown). Combined, these studies concluded that FFPE tissue can be used with confidence in future proteomics studies to discover novel disease biomarkers if the appropriate extraction technique is used. Given these convincing previous findings, FFPE tissue was used in the current study because of the much greater availability of this type of human tissue, therefore meaning that the method that we developed has much greater potential for future research.

There have been a variety of lysis methods previously suggested to increase protein identification using LC-MS on FFPE tissue. The most common methods include filter-aided sample preparation (FASP), homogenization with lysis buffers containing SDS or homogenization with commercial lysis buffers containing non-SDS detergents such as Rapigest or Liquid Tissue^2^,^37^,^43^. In this study FASP was not feasible, given the small amount of starting tissue. With regards to additional lysis methods, our results suggest that additional lysis with either an SDS containing buffer (RIPA buffer) or Rapigest didn’t significantly improve protein detection and can be omitted, hence reducing the amount of sample preparation necessary. Formic acid digestion was an additional lysis method included in this study that has not been routinely used in previous studies. It was included in this study because of its known solubilizing effect on insoluble aggregates of proteins in AD. Our preliminary data suggests that detection of a select number of other proteins (such as tau) increases after formic acid treatment, which is an important example of how using multiple lysis methods on the same sample may be beneficial in order to detect different populations of proteins.

One limitation of this method that must be considered is that even with precise LCM, there is still the possibility of collection of surrounding non-neuronal tissue. While this is an important concern and must be considered in the interpretation of results, the impact of this limitation can be minimalized by performing LCM at the highest magnification possible and using thin tissue sections to limit collection of non-neuronal tissue in the z-plane as we have done in this study. Despite this limitation, the majority of proteins detected in microdissected neurons in this study were known neuronal proteins, confirming the usefulness of this technique. It must also be considered that LCM is currently the only way to obtain a pure cell population from FFPE brain tissue and therefore the benefits of this technique outweigh the limitations.

In summary, the method we have described has great potential for any future study that wishes to perform localized proteomics using archived FFPE tissue. The major benefits of this technique include: (1) the use of human FFPE tissue, which is readily available in tissue banks around the world and is directly relevant to human disease; (2) the use of single cell populations, which increase our understanding of more specific disease mechanisms; (3) the use of LC-MS, which is a very sensitive method to quantitatively detect proteins in an unbiased manner. Importantly, the majority of experiments reported in this paper were done using tissue microdissected from a 10 year old archived tissue block, indicating that archived tissue that has been stored for many years can be used to quantify substantial numbers of proteins. Overall our strategy reduces the amount of necessary tissue for protein quantification, allows the ability to detect post-translational modifications and will facilitate the identification of novel disease associated proteins and pathways.

## Methods

### Tissue

LCM was performed using a LMD6500 microscope (Leica) on sections of temporal cortex collected from two patients with advanced AD. Informed consent for autopsy was obtained ante-mortem from subjects and post-mortem from next of kin in accordance with guidelines from the Institutional Review Board (IRB) of NYU Langone Medical Center (NYULMC). All experimental protocols were approved by the NYULMC IRB. Assessment of pathology was performed by a neuropathologist (TW) and AD pathology was graded using the “ABC” criteria, as recommended by the National Institute on Aging-Alzheimer’s Association guidelines^44^. Both patients had advanced AD pathology with ABC scores of A3B3C3. Patient characteristics are outlined in Table 2. Blocks of tissue from each of the patients were formalin fixed and paraffin embedded as part of routine autopsy procedures. 8 μm thick sections containing the temporal cortex were cut using a microtome and collected onto LCM compatible polyethylene terephthalate (PET) frame slides (Leica).

### Staining

LCM was performed on consecutive sections that were stained with cresyl violet, stained for beta amyloid using fluorescent immunohistochemistry or sections that remained unstained. Unstained sections were dewaxed and rehydrated only; sections were immersed in successive washes of xylene (2 × 30 seconds), 100% ethanol (2 × 30 seconds), 95% ethanol (30 seconds), 70% ethanol (30 seconds) and MilliQ water (30 seconds). Sections were then completely air dried for at least 12 hours prior to LCM. Sections that underwent cresyl violet staining had same dewaxing and rehydration process, followed by immersion in Cresyl Violet Acetate (0.1% in 0.3% acetic acid; Sigma) for 3 minutes, 5 dips in MilliQ water, 2 minutes in 95% ethanol and 2 × 5 minutes in 100% ethanol. Sections were then air dried in a closed container for at least 12 hours prior to LCM. For immunohistochemistry sections underwent the same dewaxing and rehydration process, followed by 3 × 5 minute washes with PBS, blocking for 1 hour at room temperature in 10% normal goat serum (Thermo) and 0.2% Triton-X in PBS and incubation with a combination of 4G8 (IBR; 1:4000) and 6E10 (IBR; 1:4000) primary antibodies in 4% normal goat serum in 0.2% Triton-X in PBS overnight at 4 °C. Sections were washed with PBS (3 × 10 min), incubated with 488 conjugated anti-mouse IgG(γ) secondary antibody (Jackson, 1:500) dissolved in 4% NGS in PBS at room temperature for 2 hours. Sections were washed with PBS (1 × 5 min), counterstained with Hoechst 33342 (Sigma; 1 μl/1 ml PBS) for 10 min, washed with PBS (3 × 5 min) and left to air dry for at least 12 hours prior to LCM.

### Laser Capture Microdissection

Regions of temporal cortex were isolated using LCM as rectangles of cortical tissue, collected at 5 X magnification. Multiple rectangles of tissue were collected totaling the desired area for each sample. Nine 10 mm^2^ samples of temporal cortex were collected to test the effect of direct in-solution digestion without detergent, direct digestion in combination with Rapigest (a mass spectrometry compatible detergent) and in-gel digestion after lysis using RIPA buffer (n = 3 for each lysis method). Three 10 mm^2^ samples were collected of temporal cortex to compare the total number of detected proteins from unstained tissue, cresyl violet stained tissue and immunostained tissue (n = 1 for each staining method). Further samples of the cresyl violet stained temporal cortex were collected containing 0.5, 1, 1.5, 2, 2.5 and 3 mm^2^ (n = 1 for each area) for the temporal cortex titration curve. Isolation of neurons from the temporal cortex using LCM was done at 40 X magnification. Neurons were identified by cresyl violet staining and automated detection of neurons was performed using AVC software (Leica). All regions/cells isolated using LCM were collected into 80 μl of MilliQ water. The time spent performing LCM depended on the type of region/cells collected, ranging between 5 minutes to 8 hours. At the end of each LCM session the collection tubes were centrifuged at maximum speed for 2 minutes and all samples were stored at −80 °C.

### Tissue digestion and LC-MS

The method was adopted from the published procedure of Alkhas *et al*.^6^. First, tissues samples were thawed and spun at 13000RPM for 2 min. 100 mM ammonium bicarbonate in 20% acetonitrile was added directly into the sample tube and then incubated at 95°C for 1 hour followed by incubation at 65 °C for another 2 hours to allow deparaffinization. The samples were subsequently processed using the following methods: 1) In-solution digestion without detergent: Samples were reduced with dithiothreitol (DTT, 20 mM) at 57 ˚C for 1 hour followed by alkylation with iodoacetamide (50 mM) at room temperature (RT) in the dark for 45 minutes. 300 ng of sequencing grade modified trypsin (Promega) was added to each cortex tissue sample and 200 ng was added to each neuron sample. Digestion proceeded overnight on a shaker at RT. The digestion was stopped by acidifying with 0.5% trifluoracetic acid (TFA). 2) In-solution digestion with RapiGest GF surfactant (Waters): After deparaffinization the supernatant was removed carefully and the tissue was reconstituted with 0.2% Rapigest solution in 50 mM ammonium bicarbonate. Samples were reduced with DTT at 57 ˚C for 1 hour (20 mM) followed by alkylation with iodoacetamide at room temperature (RT) in the dark for 45 minutes (50 mM). 300 ng of sequencing grade modified trypsin (Promega) was added to each cortex tissue sample. Digestion proceeded overnight on a shaker at RT. Samples were acidified with 0.5% TFA and incubated at 37 °C for 45 min, followed by a 10 min spin at 13000 RPM. The supernatant was transferred to new Eppendorf vial. 3) Solubilization in RIPA buffer followed by in-gel digestion: After deparaffinization the supernatant was carefully removed and incubated with 50 μl of RIPA buffer (50 mM Tris-HCL pH 7.4, 1% Triton X-100, 0.5% sodium deoxycholate, 0.1% SDS, 150 mM sodium chloride, 2 mM EDTA) containing protease inhibitor cocktail (Life Technologies). The samples were sonicated for 5 minutes with a few seconds of vortexing every minute. A 4 x LDS loading buffer (NuPAGE) was added to give a final concentration of 1 x LDS loading buffer. The sample was boiled for 5 minutes. Samples were reduced with DTT (20 mM) at 57 °C for 1 hour followed by alkylation with iodoacetamide (50 mM) at RT in the dark for 45 minutes. Samples were immediately loaded onto a NuPAGE® 4–12% Bis-Tris Gel 1.0 mm (Life Technologies Corporation) and run for approximately 5 minutes at 200 V. The gel was stained using GelCode Blue Stain Reagent (Thermo Scientific). The protein band was excised, cut into approximately 1 mm^3^ pieces and destained with a 1:1 v/v solution of methanol and 100 mM ammonium bicarbonate solution. The destained gel pieces were partially dehydrated with an acetonitrile rinse and further dried in a SpeedVac concentrator for 20 minutes. 300 ng of sequencing grade modified trypsin (Promega) was added to each gel sample. After the trypsin was absorbed 120 μl of 100 mM ammonium bicarbonate was added to cover the gel pieces. The digestion proceeded overnight on a shaker at RT. The digestion was stopped by acidifying with 5% formic acid and 0.5% TFA. 4) Formic acid extraction: After deparaffinization the sample was dried in a SpeedVac concentrator. 90 μl of 70% LC-MS grade formic acid was added to the sample, vortexed and incubated at RT overnight. The sample was sonicated three times for 3 minutes with a few seconds of vortexing in between. The sample was dried in a SpeedVac and subsequently reconstituted in 100 mM ammonium bicarbonate. Samples were reduced with DTT (20 mM) at 57 °C for 1 hour followed by alkylation with iodoacetamide (50 mM) at RT in the dark for 45 minutes. 200 ng of sequencing grade modified trypsin (Promega) was added and the digestion proceeded overnight on a shaker at RT. The samples were acidified with 5% formic acid and 0.2% TFA. For all 4 sample preparations the resulting peptides were desalted using poros beads as described previously^45^. In brief, a slurry of R2 20 μm Poros beads (Life Technologies Corporation) was added to each sample at 18:2 ratio of sample volume to poros beads. The samples were shaken at 4 °C for 3 hours and the beads were loaded onto equilibrated C18 ziptips (Millipore) using a microcentrifuge for 30 seconds at 6000 RPM. The Poros beads were further washed with 0.1% TFA followed by 0.5% acetic acid. Peptides were eluted by the addition of 40% acetonitrile in 0.5% acetic acid followed by the addition of 80% acetonitrile in 0.5% acetic acid. The organic solvent was removed using a SpeedVac concentrator and the sample reconstituted in 0.5% acetic acid. Aliquots of each sample were loaded onto a EASY spray 50 cm C18 analytical HPLC column with <2μm bead size using the auto sampler of an EASY-nLC 1000 HPLC (ThermoFisher) and solvent A (2% acetonitrile, 0.5% acetic acid). The peptides were gradient eluted into a Q Exactive (Thermo Scientific) mass spectrometer using a 2 hour linear gradient from 2% to 40% solvent B (95% acetonitrile, 0.5% acetic acid), followed by 10 minutes from 40% to 100% solvent B. Solvent B is held at 100% for another 10 minutes for column wash. The Q Exactive mass spectrometer was set up to acquire high resolution full MS spectra with a resolution of 70,000 at m/z 200, an AGC target of 1e6, with a maximum ion time of 120 ms, and scan range of 400 to 1500 m/z. Following each full MS twenty data-dependent high resolution HCD MS/MS spectra were acquired using the following instrument parameters: resolution of 17,500 at m/z 200, AGC target of 5e4, maximum ion time of 250 ms, one microscan, 2 m/z isolation window, fixed first mass of 150 m/z, and NCE of 27, dynamic exclusion 30 seconds. The MS/MS spectra were searched against the uniprot Human database (downloaded 03/28/14) using Sequest within Proteome Discoverer (ThermoFisher). The results were filtered using a <1% FDR (False Discovery Rate) searched against a decoy database and excluding proteins with less than two unique peptides.

### Interpretation of results

All proteins that were detected using LC-MS are included the in the supplementary datasheets. To prevent the detection of contaminant proteins biasing the total number of proteins identified for each sample, all non-human proteins (primarily porcine trypsin used for digestion and bovine serum albumin from staining) were removed. Compilations of previously published neuronal and AD associated proteins were generated to determine the reliability of our data. The protein lists were converted to Uniprot identifier lists and focused databases were created. The raw data were researched against these focused databases using Sequest within Proteome Discoverer to determine the number of proteins identified in both our samples and in neuronal and AD databases. The resulting list of proteins is included in the “AD or Neuronal Proteins” datasheets in each supplementary data file. This list of proteins was used to determine the percentage of neuronal and AD associated proteins in each sample.

## Additional Information

**How to cite this article**: Drummond, E. S. *et al*. Proteomic Analysis of Neurons Microdissected from Formalin-fixed, Paraffin-Embedded Alzheimer's Disease Brain Tissue. *Sci. Rep*. **5**, 15456; doi: 10.1038/srep15456 (2015).

## Supplementary Material

Supplementary Information

Supplementary Table S1

Supplementary Table S2

Supplementary Table S3

Supplementary Table S4

Supplementary Table S5

## Figures and Tables

**Table 1:** Overview of samples tested and the total number of proteins detected using LC-MS.

Type of tissue	Detailed protein list	Sample treatment	Area of tissue	Procedural replicates	Total number of proteins	Neuronal proteins (% total)	AD associated proteins (% total)
Temporal cortex region	Figure S1	In solution digest	10 mm^2^	3	1665	944 (57%)	519 (31%)
Temporal cortex region	Figure S1	Rapigest	10 mm^2^	3	1773	984 (55%)	528 (30%)
Temporal cortex region	Figure S1	RIPA buffer In gel digest	10 mm^2^	3	1598	914 (57%)	505 (32%)
Temporal cortex region (unstained)	Figure S2	In solution digest	10 mm^2^	1	846	544 (64%)	373 (44%)
Temporal cortex region (cresyl violet stained)	Figure S2	In solution digest	10 mm^2^	1	955	601 (63%)	397 (42%)
Temporal cortex region (immunostained)	Figure S2	In solution digest	10 mm^2^	1	1009	636 (63%)	410 (41%)
Temporal cortex region	Figure S3	In solution digest	0.5 mm^2^	1	649	456 (70%)	327 (50%)
Temporal cortex region	Figure S3	In solution digest	1 mm^2^	1	711	492 (69%)	339 (48%)
Temporal cortex region	Figure S3	In solution digest	1.5 mm^2^	1	856	566 (66%)	388 (45%)
Temporal cortex region	Figure S3	In solution digest	2 mm^2^	1	956	615 (64%)	406 (42%)
Temporal cortex region	Figure S3	In solution digest	2.5 mm^2^	1	970	625 (64%)	409 (42%)
Temporal cortex region	Figure S3	In solution digest	3 mm^2^	1	949	613 (65%)	395 (42%)
Temporal cortex neurons	Figure S4	In solution digest	0.5 mm^2^	1	202	159 (79%)	109 (54%)
Temporal cortex neurons	Figure S4	In solution digest	1 mm^2^	1	261	203 (78%)	143 (55%)
Temporal cortex neurons	Figure S4	In solution digest	1.5 mm^2^	1	399	306 (77%)	209 (52%)
Temporal cortex neurons	Figure S4/S5	In solution digest	2 mm^2^	1	433	337 (78%)	233 (54%)
Temporal cortex neurons	Figure S4	In solution digest	2.5 mm^2^	1	396	306 (77%)	199 (50%)
Temporal cortex neurons	Figure S5	Formic acid -In solution digest	2 mm^2^	1	503	387 (77%)	254 (50%)

Detailed protein identification results can be found in the supplementary data sheets. The percentages of identified proteins that were confirmed neuronal proteins or known to be associated with AD are shown in the final two columns. For these results the more stringent neuronal database was used, ensuring that only proteins that have been previously confirmed in published studies were classified as neuronal.

**Table 2:** Patient characteristics.

Patient	Experiment	Tissue block storage time	Diagnosis	Gender	Age
1	Staining comparison	2 years	AD (A3,B3,C3)	M	82
2	Lysis comparison, temporal cortex titration curve, neuron titration curve, neurons (formic acid extraction)	10 years	AD (A3,B3,C3)	F	85
