# Supplementary material for: Proteomic analysis of neurons microdissected from formalin-fixed, paraffin-embedded Alzheimer’s disease brain tissue
Source: Sci Rep. 2015 Oct 21;5:15456. doi: 10.1038/srep15456 (PMC4614382; doi:10.1038/srep15456)
Supplement: Supplementary Information [file srep15456-s1.pdf]

Manuscript Title:

Proteomic Analysis of Neurons Microdissected from Formalin-fixed, Paraffin-Embedded Alzheimer's Disease Brain Tissue

Author List:

Eleanor S Drummond[1], Shruti Nayak[2], Beatrix Ueberheide[2], Thomas Wisniewski[3*]

Supplementary Legend for Datasets 1-5:

Supplementary Dataset S1 – Lysis methods: Comparison of detected proteins from (A) In solution direct digestion, (B) In solution direct digestion in combination with Rapigest and (C) In gel digestion after RIPA buffer lysis. GO annotation is included for each identified protein detailing molecular function, biological process and cellular function. Detected proteins datasheet identifies all proteins detected (including contaminants), complete with identified peptide data. The neuronal or AD associated datasheet contains all identified proteins without the individual peptide data. All non-human contaminant proteins have been removed and each protein that has previously been confirmed to be present in neurons or associated with AD is identified.

Supplementary Dataset S2 – Staining methods: Comparison of detected proteins from (A) unstained, (B) immunostained (IHC) and (C) cresyl violet stained tissue.

Supplementary Dataset S3 – Temporal cortex titration curve: Comparison of detected proteins in (A) $0.5mm^2$, (B) $1mm^2$, (C) $1.5\ mm^2$, (D) $2mm^2$, (E) $2.5mm^2$ and (F) $3mm^2$ areas of temporal cortex.

Supplementary Dataset S4 – Neuron titration curve: Comparison of detected proteins in (A) $0.5mm^2$, (B) $1mm^2$, (C) $1.5\ mm^2$, (D) $2mm^2$ and (E) $2.5mm^2$ combined areas of neurons isolated using LCM.

Supplementary Dataset S5 – Neuronal proteins (formic acid digestion): Comparison of detected proteins in $2mm^2$ combined area of neurons isolated from temporal cortex (A) digested with formic acid treatment and (B) digested without formic acid treatment.

Common legend for all supplementary datasets:

- **Score:** Sum of the scores of the individual peptides.
- **Coverage:** Percentage of the protein sequence identified in this analysis.
- **#peptides:** Number of distinct peptide sequences identified per protein per protein group.
- **#PSM** (Peptide Spectrum Matches): Total number of identified peptide sequences for the protein, including those redundantly identified.
- **AA:** Represents the total number of amino acids in the protein sequenc.
- **MW [kDa]:** Molecular weight of the protein.
- **Calc PI:** Calculated isoelectric point, which is the PH at which a particular molecule carries no net charge.
- **Molecular functions:** Gene Ontology categories of protein's molecular functions.
- **Cellular component:** Gene Ontology categories of the protein's cellular component
- **Biological process:** Gene Ontology of the protein's biological processes.

Legend for the peptide identification results.

- **High confidence:** Confidence level of peptide identification. We included only high confidence peptides based on a better than 1% FDR searched against a decoy database.
- **# Proteins:** Number of proteins in which the peptide is found.
- **#Protein Groups:** Number of protein groups in which the peptide is found.
- **Accession number:** Unique identifier of all protein groups that include this peptide sequence.
- **Modifications:** We include Carbamidomethylation of cysteines which is a result of the reduction and alkylation reactions, Deamidation of N, Q amino acids, Oxidation of M. For samples treated with formic acid we included Formylation of lysine.
- **MH+[Da]:** Monoisotopic mass of the peptide ions in Daltons.
- **Xcorr:** Number of fragment ions that are common to two different peptides with the same precursor mass and calculates the cross-correlation score for all candidate peptides queried from the database.
- **qValue:** Minimal false discovery rate at which the identification is considered incorrect.
- **PEP**(Posterior Error Probability)**:** Probability that the observed PSM is incorrect.

Missed cleavages: Number of cleavage site in a peptide sequence that an enzyme failed to cleave.
